# The SUMO System and TGFβ Signaling Interplay in Regulation of Epithelial-Mesenchymal Transition: Implications for Cancer Progression

**DOI:** 10.3390/cancers10080264

**Published:** 2018-08-08

**Authors:** Ayan Chanda, Anusi Sarkar, Shirin Bonni

**Affiliations:** Department of Biochemistry and Molecular Biology, The Arnie Charbonneau Cancer Institute, University of Calgary, Calgary, AB T2N 4N1, Canada; achanda@ucalgary.ca (A.C.); anusi.sarkar@ucalgary.ca (A.S.)

**Keywords:** SUMOylation, TGFβ, EMT, cancer

## Abstract

Protein post-translational modification by the small ubiquitin-like modifier (SUMO), or SUMOylation, can regulate the stability, subcellular localization or interactome of a protein substrate with key consequences for cellular processes including the Epithelial-Mesenchymal Transition (EMT). The secreted protein Transforming Growth Factor beta (TGFβ) is a potent inducer of EMT in development and homeostasis. Importantly, the ability of TGFβ to induce EMT has been implicated in promoting cancer invasion and metastasis, resistance to chemo/radio therapy, and maintenance of cancer stem cells. Interestingly, TGFβ-induced EMT and the SUMO system intersect with important implications for cancer formation and progression, and novel therapeutics identification.

## 1. Introduction

Response to intrinsic and extrinsic cues is a hallmark of living cells. Cells need to respond to diverse signals using a limited set of molecular components of which proteins form a major cellular constituent [1]. Reversible modifications of proteins, also known as post-translational modifications, by regulating various properties of these proteins including stability, interaction with other biomolecules and subcellular localization, can have important functional consequences for cellular responses to stimuli [2]. Identification, characterization, and mapping of proteins’ modifications including phosphorylation, glycosylation and ubiquitination, to specific amino acid residues on target proteins are critical in understanding functional significance of such modifications in a biological context [3,4]. 

SUMOylation is a post-translational modification that is related to ubiquitination. Increasingly, it is becoming clear that SUMOylation can affect a wide array of biological responses during development and homeostasis including cell differentiation, apoptosis and senescence [5]. Modification by SUMO is found in all eukaryotes [6]. SUMOylation culminates in an isopeptide bond formation between the C-terminal carboxyl group of the protein Small Ubiquitin-like Modifier (SUMO) and ε-amino group of a lysine residue on a specific protein substrate [7]. The mammalian SUMO family comprises SUMO1 to SUMO5 ranging in length from 92 to 97 amino acid residues [7]. SUMO1 shows 50% identity to each of SUMO2 and SUMO3; SUMO2 has 95% identity with SUMO3, while SUMO4 shows 87% homology to SUMO2 [7,8]. The most recently identified SUMO member SUMO5 has been reported to show tissue-specific expression in primates including testes and blood cells [9]. The SUMOylation machinery is mostly restricted to the nucleus [10]. Hendriks and Vertegaal have curated data from several studies and found that 18% of the human proteome, which corresponds to approximately 3700 human proteins, is targeted by the SUMO machinery [11]. Hence, understanding the biochemical and biological significance of SUMOylation in living organisms has been the subject of numerous studies [5,11,12].

## 2. The SUMOylation Machinery

**Figure 1:**
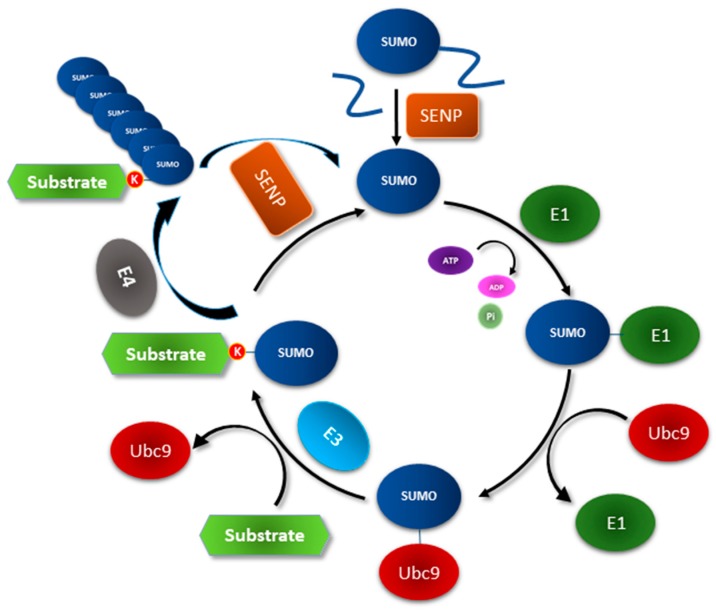
The Small Ubiquitin-like Modifier (SUMO) conjugation system. Members of the sentrin specific protease (SENP) family of endopeptidases can cleave pro-SUMO into a C-terminal peptide and the mature form of SUMO, revealing the C-terminal diglycine motif. With the help of ATP, the SUMO E1 activating enzyme then forms a thioester bond with SUMO's C-terminal carboxyl group of the diglycine motif. The SUMO E2 conjugating enzyme Ubc9 next forms a thioester bond with activated SUMO. SUMO-conjugated Ubc9 binds a substrate and transfers the SUMO group to a specific lysine residue(s) within the substrate. A SUMO E3 ligase by binding to Ubc9 and a specific substrate can promote the transfer of SUMO from Ubc9 to the substrate. A SUMO E4 elongase may promote the sequential conjugation of SUMO molecules to specific lysine residues within SUMO molecules, starting with the one forming the isopeptide bond with the substrate, leading to poly-SUMOylation of the substrate. SENPs can bind to mono and poly-SUMOylated substrates leading to isopeptide bond cleavage and release of deSUMOylated substrates and individual SUMO molecules to the SUMO pool to be re-utilized by the SUMO system.

SUMOylation is a multistep biochemical process. In the first step, SUMO is activated in an ATP-dependent manner by a SUMO E1 activating enzyme, which is a heterodimeric complex consisting of SUMO Activating Enzyme (SAE) 1 and the adaptor/regulator subunit SAE 2. Once activated, SUMO is transferred to a SUMO E2 conjugating enzyme, which in mammals is represented by the protein Ubiquitin Carrier 9 (Ubc9). SUMO is then conjugated to specific lysine residues within a substrate by Ubc9. A SUMO E3 ligase binds to specific substrates as well as to Ubc9, and in this way helps target these proteins for SUMOylation. SUMOylation is a reversible process due to the action of specific deSUMOylases called sentrin specific proteases (SENPs). SENPs also promote the maturation of the SUMO precursor protein [13,14]. Under certain conditions, several SUMO molecules may get conjugated to each other via isopeptide bonds to form poly-SUMOylated chains [15]. A recent structure-based study has suggested the presence of a special class of SUMO enzymes in vertebrates which is termed SUMO E4 elongases as its members are found to be essential for SUMO2/3 chain elongation but not for these SUMOs attachment to a substrate [16] (Figure 1). SUMO monomers or chains have an affinity to specific motifs called SUMO interacting motifs (SIMs), which are characterized by a stretch of negatively charged amino acids or phosphorylated serine/threonine residues followed by a hydrophobic core sequence. The hydrophobic stretch in SIM associates with the alpha-helix and beta2-strand surfaces of the SUMO proteins, while the negatively charged residues in SIM specify interaction with distinct SUMO isoforms [17]. SIMs have been identified in different types of proteins including SUMO E3 ligases, transcription factors, and transcriptional coactivators or corepressors [11]. SUMOylation is a very tightly regulated process and perturbation may lead to disease conditions including diabetes, cardiovascular disease and cancer [13].

A SUMO E3 ligase, which can contribute to substrate recognition and targeting by the SUMO pathway, is the most dynamic component of the SUMOylation cascade [7]. A number of SUMO E3 ligases contain SIM motifs and really interesting new gene (RING) finger domains which have been suggested to promote the interaction of a SUMO E3 ligase with SUMO and Ubc9, respectively [18]. However, how SUMO E3 ligases recognize SUMO substrates still remains largely unknown. The Protein Inhibitor of Stats (Signal Transducer and Activator of Transcription) (PIAS) family of SUMO E3 ligases proteins is a widely-studied class of SUMO E3 ligases. The nuclear pore protein RanBP2, polycomb group protein Pc2, and tripartite motif-containing (TRIM)-containing proteins represent other types of SUMO E3 ligases [7,18,19,20,21].

Controlled SUMOylation is important for normal cellular functions and dysregulation can be involved in pathophysiological conditions [12,22]. Alterations in the status of SUMOylation of a given protein may serve as an ON/OFF switch for the target protein’s biochemical/physiological function. SUMOylation could have important implications for control of protein activity and cellular fate. Biologics or chemicals that can either suppress or promote SUMOylations of specific protein substrates, however, have remained elusive.

## 3. Epithelial-Mesenchymal Transition (EMT)

EMT is a key cellular process that allows cells to escape the local environment and migrate to secondary sites during development and disease [23]. During EMT, epithelial cells transdifferentiate to become more migratory, invasive, and mesenchymal-like [23]. The process of EMT consists of three broad interconnected sets of events which may occur in parallel. The first set of events leads to weakening or loss of cell-cell contact and apical-basal polarity features of epithelial tissues. The second set of actions promotes cortical to stress-fiber actin cytoskeletal reorganization which results in cuboidal to fibroblastic like cell shape change with increased cell motility and invasiveness. The third set of events involve changes in gene expression signature from epithelial to mesenchymal type, where expression of epithelial markers including E-cadherin is repressed, whereas expression of mesenchymal markers including N-cadherin is upregulated. Master transcriptional regulators (EMT-TF) including Snail/Snai1, Slug/Snai2, ZEB1, ZEB2/SIP1, and Twist drive the EMT gene expression program and invasive behaviour of cells [24,25,26,27]. These EMT-TFs recruit DNA-and chromatin-remodelling enzymes to gene promoters and regulators to suppress the expression of epithelial cell-specific genes including E-cadherin, claudins and cytokeratins, and promote the expression of mesenchymal genes like N-cadherin, fibronectin and matrix-metalloproteinases (MMPs) [25]. 

EMT, a fundamental process in embryogenesis, can be re-triggered in cancer and has been related to tumor progression [28,29]. EMT-related changes in tumor cells allow these cells to escape from the primary sites, enter the circulation, and then move out to invade distant sites where secondary tumors and hence metastases may arise. Interestingly, EMT may also preferentially enrich for cancer stem cells (CSCs), which have been implicated in primary tumor and metastasis formation [30,31]. CSCs are defined as tumor cells with self-renewal and tumorigenic properties [32]. Importantly, EMT and CSCs have been implicated in tumor recurrence following chemotherapy and radiotherapy due to increased survival and evasion of cell death [26,33]. The secreted protein, transforming growth factor β (TGFβ) is a potent inducer of EMT during development and cancer [34]. There has been a great deal of scientific inquiries to discover and characterize regulators of TGFβ-induced EMT in the context of development and cancer.

## 4. TGFβ Signaling Pathway

**Figure 2:**
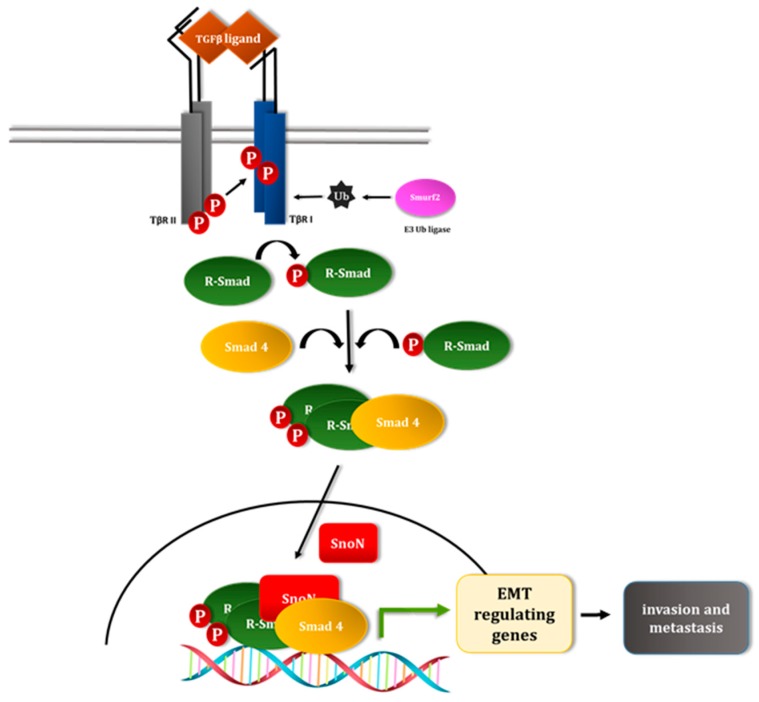
The transforming growth factor beta (TGFβ)-smad signaling pathway. The TGFβ ligand binds the transmembrane type II ser/thr kinase receptor (TβRII) leading to recruitment of type I ser/thr kinase receptor (TβRI). Within this complex, TβRII transphosphorylates the TβRI within the GS domain which in turn promotes the activation of the TβRI's kinase moiety. The activated ligand-receptor heteromeric complex associate with the Receptor-regulated Smad 2 and 3, whereby the last two C-terminal serine residues within these Smads are targeted by phosphorylation by the TβRI kinase. The TGFβ-phosphorylated R-Smad dissociates from the receptor complex and forms a hetero-oligomer complex with the common-partner Smad4, and the complex accumulates in the nucleus. Within the nucleus, the Smad complex binds to specific DNA elements on TGFβ-responsive genes and in collaboration with other transcription factors and transcriptional coregulators, including SnoN, can positively or negatively regulate the expression of these genes and consequent responses including EMT. The ubiquitin E3 ligase Smurf2 is recruited to the activated TGFβ receptors leading to their ubiquitin-mediated degradation and suppression of TGFβ signaling pathway.

TGFβ plays pleiotropic and critical roles in the developing and adult organisms [35]. TGFβ can also contribute to disease progression including organ fibrosis and cancer [36,37]. The canonical Smad pathway plays a major role in mediating TGFβ signal from the cell surface to the nucleus. TGFβ ligands bind to the TGFβ type II serine/threonine kinase receptors (TβRII) on the cell surface, which in turn recruit and trans-phosphorylate the TGFβ type I serine/threonine kinase receptors (TβRI/ALK5) at multiple serine and threonine residues within the glycine-serine rich (GS) domain [38]. Phosphorylated GS region of TβRI in turn promotes the activation of the TβRI kinase moiety, leading to recruitment and C-terminal SXS motif phosphorylation of Receptor-regulated Smad2 (R-Smad2) and R-Smad3 [38,39]. The phosphorylated R-Smad2/3 (pSmad2/3) binds Smad4 and the complex accumulates in the nucleus. Once in the nucleus, the R-Smad-Smad4 complex binds to specific DNA elements on TGFβ-target genes and in conjunction with diverse transcription factors and transcriptional coregulators regulates TGFβ-responsive gene expression in a cell and context specific manner [38,40,41] (Figure 2). Inhibitory Smads (Smad6 and Smad7) block the TGFβ-Smad pathway by different mechanisms including by competing with the R-Smads for receptor binding or via recruitment of the HECT-containing E3 ubiquitin ligase Smurf2 to the activated receptors thus targeting them for ubiquitin-mediated degradation (refer Section 5.3) [42]. TGFβ may also signal, in a cell and/or context-dependent manner, via non-Smad pathways independently or in collaboration with the Smad pathway with important consequences for a wide array of cellular responses including EMT [35,41,43]. Understanding the mechanisms that regulate TGFβ signaling axes may provide critical insights into how tissue and organ morphogenesis are controlled during development with important implications for the understanding of disease progression.

The SUMO pathway targets diverse components of the TGFβ signaling pathways. This review provides an overview of the literature on SUMOylation of specific mediators and regulators of the TGFβ signaling pathways, and the functional impact of the SUMO system on the ability of these substrates to affect TGFβ-regulated transcriptional and biological responses, with a special focus on EMT induction.

## 5. SUMOylation of TGFβ Pathway Signal Transducers

### 5.1. TGFβ Receptor

**Figure 3:**
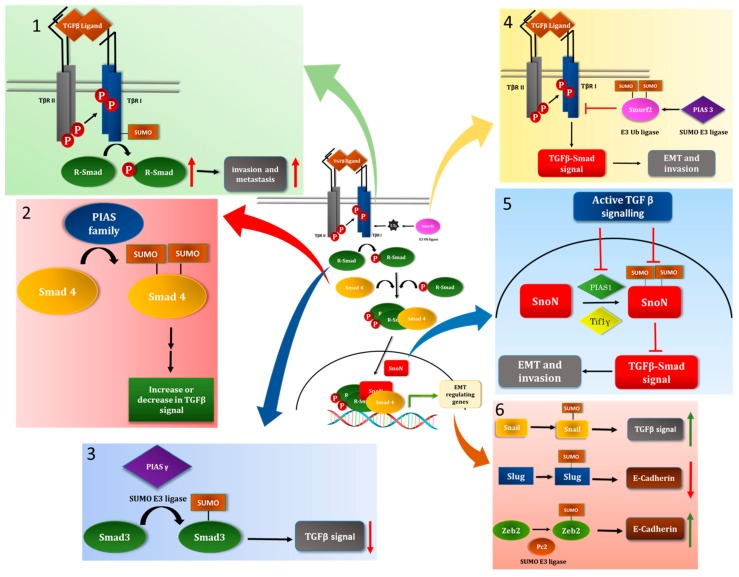
SUMOylation system and TGFβ-signaling interplay in the context of induction of epithelial-mesenchymal transition (EMT). Six schematic models summarizing how the SUMO system and TGFβ pathway collaborate or antagonize each other in controlling transcriptional responses that are critical for EMT induction. Briefly, multiple components of the TGFβ signaling axis (in centre) as well as EMT-inducing transcription factors (EMT-TFs) can be targeted by the SUMO system with diverse consequences for EMT-related events. TGFβ signaling has also been shown to regulate the ability of the SUMO system in targeting such substrates. (1) TβRI as a target of the SUMO system: Lysine residues downstream of kinase domain of TβRI are targets of SUMOylation which can promote the receptor kinase activity and downstream signaling events including R-Smad phosphorylation and transcriptional activity [44]. (2) Smad4 is a target of SUMOylation: The SUMO system targets two Lysine residues in Smad4 for SUMOylation that may lead to diametrically opposite effect on TGFβ signaling depending on cell type and context [50,51,52,53]. (3) The SUMO system and Smad3: Studies have suggested that Smad3 is SUMOylated, which suppresses Smad3's ability to mediate TGFβ signaling [55]. (4) The HECT-containing ubiquitin E3 ligase Smurf2 as a target of the SUMO pathway: Ubc9, promoted by PIAS3, targets specific lysine residues within Smurf2 for SUMOylation. SUMOylation promotes the ability of Smurf2 to reduce the protein abundance of TβRI. Overall, SUMOylation promotes the ability of Smurf2 to suppress TGFβ-Smad-induced EMT [56,57]. (5) The transcriptional coregulator SnoN and the SUMO system: Two distinct SUMO E3 ligases, PIAS1 and TIF1γ, promote the SUMOylation of both Lysine residues 50 and 383 on SnoN. SUMOylation is critical for the ability of SnoN to suppress TGFβ-induced EMT. In order to induce EMT, TGFβ signaling suppresses SnoN SUMOylation at least in part by increasing the protein turnover of its SUMO E3 ligase PIAS1 [14,58,59]. (6) SUMOylation regulates the activity of several EMT inducing transcription factors: TGFβ-induced expression of Snail, Slug, and Zeb2 contribute significantly to EMT induction. Snail, Slug and Zeb2 are targets of the SUMO system. SUMOylation promotes the ability of Snail and Slug to induce EMT, while suppressing Zeb2 role in EMT progression [60,61,62].

Kang et al reported that TβRI/ALK5 is SUMOylated and identified Lysine 389 (K389) as a major site of SUMOylation on the receptor. Interestingly, modification by SUMO appeared to be unique to TβRI/ALK5, as other members of the TGFβ superfamily-activated type I serine/threonine kinase receptors failed to show modification by SUMO, which was consistent with the lack of a SUMO consensus motif in these receptors. Lysine 389 is located downstream of the kinase domain of TβRI/ALK5. SUMOylation of TβRI has been suggested to be critical for TβRI/ALK5 binding and catalyzing the phosphorylation of R-Smads, and hence TGFβ-Smad-dependent gene expression. Interestingly, SUMOylation of TβRI/ALK5 was suggested to be important for TGFβ-induced invasion and lung metastasis of Ras-transformed fibroblasts. The kinase activities of both TβRI/ALK5 and TβRII were reported to be required for TβRI/ALK5 SUMOylation to occur, suggesting a dependence of SUMOylation on phosphorylation [44] (Figure 3). Phosphorylation of a serine/threonine residue within an extended SUMO consensus motif has been suggested to promote SUMOylation at the lysine residue within the consensus motif [45]. However, that TβRII-mediated phosphorylation of TβRI occurs at the GS region which is ~200 amino acids upstream of Lysine 389 may suggest a distinct mode of regulation of SUMOylation [44]. Interestingly, TβRI/ALK5 S387Y alleles have been found to be enriched in tissues derived from distant metastases, and not primary tumors, of human breast and head and neck cancers [46,47]. Counterintuitively, the authors observed that mutation of Serine 385 to tyrosine (S385Y) suppresses TβRI SUMOylation and metastatic growth of MEFs when compared to wild type protein [44]. The dichotomy between these two studies [44,46] may be explained by the dual nature of TGFβ signaling as a tumor suppressor in early stages and as a tumor promoter in later stages of carcinogenesis [37]. Overall, future studies should identify the SUMO E3 ligases and SENPs that regulate TβRI SUMOylation as well as the functional relevance of this modification.

### 5.2. Members of the Sma-Mad (Smad) Family of the Signal Transducers

The R-Smads and the common-partner Smad4 proteins possess two conserved globular domains called the Mad homology 1 (MH1) and the MH2 domains, located N-terminally and C-terminally, respectively, and are linked together by a linker region. Inhibitory Smads have an MH2 but lack the MH1 domain. The β-hairpin region found in MH1 domain in Smad4 and some of R-Smads recognize specific DNA elements called Smad binding elements (SBEs), whereas the MH2 domain largely confers interaction of the Smads with other proteins including other Smads and TGFβ superfamily receptors [48]. The linker region is important for the subcellular localization of the Smad proteins [49].

Smad4 is a SUMO substrate. In particular, Lysines 113 and 159, located in MH1 domain and linker region of Smad4, respectively, represent major sites of SUMOylation in Smad4 [50,51,52,53]. Smad4 appears to associate via its MH1 domain with Ubc9 [51]. The role of SUMOylation on the ability of Smad4 to mediate TGFβ signaling might be cell and context-dependent. Mutation of Lysines 113 and 159 into arginine residues, thus leading to loss of SUMOylation, was reported to enhance Smad4-induced TGFβ-induced transcriptional responses in human breast and colon cancer cells, as well as in developing *Xenopus* embryo cells suggesting that SUMOylation counteracts Smad4’s ability to mediate TGFβ signaling [50,52]. However, the single or combined overexpression of SUMO and Ubc9, together with that of wild type Smad4 were found to promote Smad4-dependent TGFβ-induced transcription in the human breast and colorectal carcinomas [50,51]. Consistent with a promoting role of SUMOylation in Smad4-mediated signaling, overexpression of SUMO1, Ubc9, alone or together has been reported to promote the nuclear retention and protein stability of Smad4 [50,54]. Interestingly, in the same aforementioned study, it was reported that overexpressed SUMO-Smad4 fusion protein or co-overexpressed SUMO1 and Ubc9 suppressed TGFβ-induced transcription in COS-7 monkey kidney fibroblast-like cells, suggesting a potential negative role for SUMOylation in the ability of Smad4 to mediate TGFβ-responses in this cell type [52] (Figure 3). Further studies would help to decipher whether SUMOylation promotes or suppresses the ability of Smad4 to mediate TGFβ signaling and responses, and if SUMOylation-dependent effects may be related to factors such as cell type and context.

Different members of the PIAS family of SUMO E3 ligases have been suggested to act as SUMO E3 ligases for Smad4 [50,52,53]. Whether distinct members of the PIAS family act more selectively than others to associate with and promote Smad4 SUMOylation requires additional detailed studies.

Studies also suggest an interplay between TGFβ signaling and the SUMOylation system in regulation Smad4 modification by SUMO. Thus, it has been suggested that activation of TGFβ signaling pathways promotes Smad4 SUMOylation in a p38 MAPK-dependent but R-Smad-independent manner. Interestingly, in this study it was suggested that activation of p38 signaling axis promotes the expression of PIASxβ, and potentially Smad4 SUMOylation. Coexpression of PIASxβ with SUMO1, Smad2 and Smad4 led to significant increase in TGFβ-responsive 3TP-luciferase and GAL4-luciferase reporter activities as compared to expression of only Smad2 or Smad4 in COS-7 cells [53].

The implication of SUMOylation in Smad4 role in diseases has been investigated. Zhou et al found that high glucose condition in diabetic nephropathy leads to an increase in Smad4 SUMOylation in mesangial cells which correlated with TGFβ-induced gene transcription and pathological effects [63]. Overexpression of PIAS1, with vector only, or with wild type or SUMO loss of function mutant Smad4, in rat hippocampi in vivo supported the idea that Smad4 SUMOylation on Lysines 113 and 159 in rodent hippocampus promotes gene expression of the skeletal myopathy gene tropomyosin 2 (TPM2), and improved memory formation and spatial learning [64]. Intriguingly, in the hFOB1.19 osteoblast cells, Smad4 appears to be conjugated by SUMO2/3 and not SUMO1. Importantly, SUMO2/3 conjugation was found to promote the ability of Smad4 to suppress oxidative stress-induced apoptosis in osteoblasts, thus inhibiting the progression of osteoporosis in mice [65]. 

PIAS3 has been reported to interact more specifically with Smad3 and Smad2 than with Smad4 using coimmunoprecipitation analyses. In addition, PIAS3 was found to promote in a RING-domain-dependent manner, Smad-induced transcription of TGFβ-responsive genes in the HaCaT human skin keratinocytes. This effect of PIAS3 was suggested to be independent of its ability to promote Smad4 SUMOylation. Instead, it was suggested that PIAS3 promotes a ternary complex involving PIAS3, Smad3 and the HAT p300 leading to TGFβ-induced gene transcription [66]. In contrast, findings from a recent study using a three-dimensional culture model suggested that PIAS3 promotes the SUMOylation of the HECT-containing ubiquitin E3 ligase Smurf2 with important implications for suppression of TGFβ-induced EMT and invasiveness in non-transformed mammary epithelial cells and breast carcinoma, respectively (see below) [56,57]. 

In another study by Imoto et al., all members of the Smad family of signal transducers were found to interact via their MH2 domain with the SUMO E3 ligase PIASγ (also termed PIAS4). However, in vivo SUMOylation assays in COS-7 cells suggested that only Smad3 was SUMO-modified by PIASγ. In addition, overexpression of PIASγ appeared to significantly reduce TGFβ-Smad3-responsive reporter activity in COS-7 and hepatoma Hep3B cells. Stimulation of cells with TGFβ led to a significant increase in PIASγ protein abundance, possibly in a negative feedback loop, and exogenous expression of PIASγ suppressed TGFβ-induced PAI-1 gene expression [55] (Figure 3). The finding that PIASγ suppresses TGFβ signaling is consistent with data suggesting that PIASγ acts as a SUMO E3 ligase for Smad4 and in this manner may suppress TGFβ signaling [52]. Whether PIASγ acts as a SUMO E3 ligase for Smad3 and Smad4 simultaneously, and what is the cumulative effect if any, need further analyses. 

That the Smads are SUMO targets with implication for regulation of TGFβ-mediated signaling and responses raises the question regarding the mechanism by which SUMOylation regulates Smad function. One possibility could be explained by the formation of PIAS-Smad3/4 ternary complex, e.g., involving PIASγ, leading to recruitment of HDAC1 to repress TGFβ-induced transcriptional responses [67]. Whether PIASγ promotes SUMOylation of Smad3 and/or Smad4 which in turn can recruit non-SUMOylated proteins to form a higher order protein complex remains to be investigated.

Overall, Smad3 and Smad4 appear to be SUMO targets with functional relevance for the roles of these Smads in mediating TGFβ signaling. How Smad3 or Smad4 SUMOylation is regulated remains largely unknown. The diametrically opposite effects of SUMOylation of these Smads on TGFβ-responsive elements that have been reported also awaits further scrutiny. PIAS3 and PIASγ have diverse sets of substrates, thus it will be interesting to characterize the role and mechanisms of such PIAS3/PIASγ-substrate(s) axis in TGFβ signaling and responses. 

### 5.3. The E3 Ubiquitin Ligase Smurf2 as a SUMO Substrate

Smurf2, or SMAD Ubiquitination Regulatory Factor 2, is a HECT-containing E3 ubiquitin ligase which was identified following the discovery of Smurf1 gene product via Smad1-interactome screen [68]. Smurf2 promotes the ubiquitination and consequent degradation of protein substrates [69]. Smurf2 has been suggested to target diverse sets of proteins for ubiquitination including components and regulators of the TGFβ signaling pathway [69,70,71,72]. Overall, Smurf2 appears to have diverse and sometimes opposing effects on biological processes including EMT raising the question of the mechanisms that regulate Smurf2 functions. Whether Smurf2 is a target of post-translational modification remained largely unknown. Remarkably, recent evidence suggests that Smurf2 is a SUMO pathway substrate. In particular, Lysine residues 26 and 369 are major sites of SUMOylation on Smurf2. The SUMO E3 ligase PIAS3 associates with and promotes the SUMOylation of Smurf2. Expression of the deSUMOylases SENP1 and SENP2 but not SENP3 inhibited Smurf2 SUMOylation suggesting that SENP1 and SENP2 might be deSUMOylases for Smurf2 [56]. 

**Figure 4:**
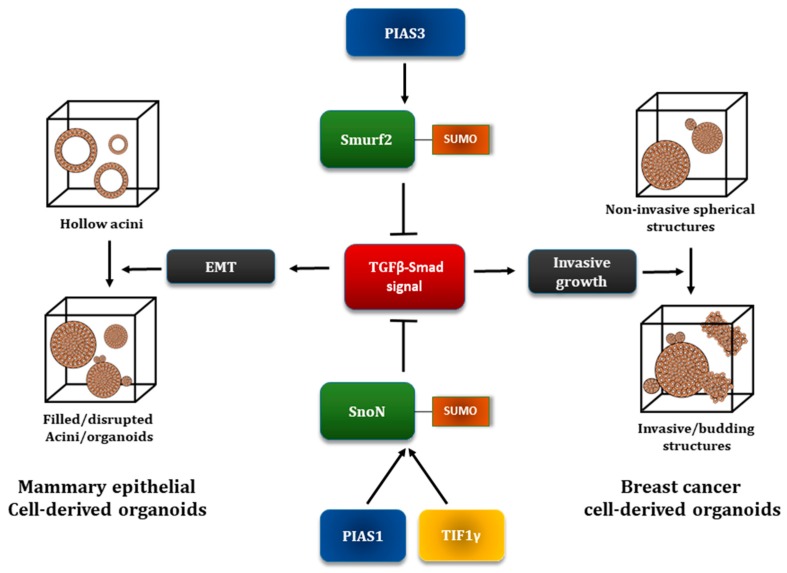
Three-dimensional (3D) culture system as a model to study EMT and invasive growth. The 3D culture system has been suggested to better mimic the in vivo system, as compared to a conventional 2D culture. This system also provides a robust cellular system to capture morphological changes in response to different cellular events including EMT. Isolated epithelial cells, e.g., NMuMG cells, when cultured in the context of a 3D-matrix, proliferate and assemble into multicellular structures (or organoids) characterized by hollow spheres or acini. Increased TGFβ signaling, and EMT induction can manifest as acinar filling, buddings and a key hallmark of EMT (not shown here)-loss/mislocalization of the epithelial cell marker E-cadherin. Isolated breast cancer cells, e.g., the TNBC MDA-MB-231 cells, can form filled solid spheroids with sometimes invasive edges. TGFβ-induced EMT can manifest as disruption and invasive growth of these organoids. The SUMO system is important for the ubiquitin E3 ligase Smurf2 and the transcriptional coregulator SnoN to suppress TGFβ-induced EMT-like phenotypes in the 3D-mammary epithelial (NMuMG) and breast cancer (MDA-MB-231) cell-derived organoids as manifested by acinar filling and invasive growth, respectively [14,56,57,58,59].

The functional consequences of SUMOylated-Smurf2 in TGFβ-induced EMT has been investigated using a Three-Dimensional (3D)-mammary epithelial cell-derived organoids system [56]. As a preamble, gland-derived non-transformed epithelial cells when cultured in the context of an extracellular matrix, e.g. Matrigel, which provides a microenvironment resembling the in vivo environment, proliferate and form multicellular structures or organoids characterized by hollow centres or acini. 3D-epithelial cell-derived acini provide a robust model to follow morphological alteration like those induced by processes such as EMT [73]. In particular, EMT inducing signals, such as TGFβ, promote filling of the hollow centre, budding and invasive behaviour of the organoids (Figure 4). These TGFβ-induced morphological alterations in the epithelial cell-derived organoids are accompanied by decrease or mislocalization of E-cadherin and reorganization of actin from cortical to stress fiber-like [56].

Loss and gain of function of Smurf2 analyses suggested that Smurf2 suppresses the ability of TGFβ to induce EMT in the non-transformed mouse NMuMG mammary epithelial cell-derived organoids. Interestingly, a SUMO loss of function Smurf2 mutant in which Lysine residues 26 and 369 were converted to Arginine (Smurf2(KdR)) promoted EMT, even in the absence of TGFβ stimulation suggesting that SUMOylation is important for the ability of Smurf2 to suppress EMT (Figure 3 and Figure 4). Mechanistically, it was found that SUMOylation significantly enhances the ability of Smurf2 to reduce the protein abundance of TβRI [56]. 

Three-dimensional-transformed carcinoma cell-derived organoids, for example organoids derived from human triple negative breast cancer MDA-MB-231 cells, can display filled spherical structures with some degree of outward protrusions and budding. TGFβ promotes the invasive growth of these organoids, where multicellular structures show extensive budding, deformation, and invasive behaviour [74]. In a recent study, it was found that PIAS3 acts at least in part via SUMOylation of Smurf2 to suppress the invasive growth of breast cancer cell-derived organoids suggesting a potential anti-metastatic activity of SUMOylated-Smurf2 [57] (Figure 3 and Figure 4). The PIAS3 anti-invasive effect is also consistent with translational findings suggesting that expression of PIAS3 correlates with reduced metastasis of multiple tumor types [75]. Whether the effect of PIAS3 in suppressing TGFβ signaling is context or cell-type dependent needs further investigation as the literature suggests that PIAS3 promotes Smad2/3-dependent TGFβ transcriptional responses, although the necessity of E3 ligase activity of PIAS3 was not evaluated in this context [66]. 

### 5.4. The Transcriptional Coregulator SnoN as a Target of the SUMO Pathway

SnoN, or Ski-related novel protein N, is a key component of the TGFβ signaling pathway [14,76,77,78]. Initial studies suggested that SnoN acts as a negative regulator of TGFβ-Smad signaling. However, it is clear now that SnoN can positively or negatively regulate TGFβ signaling with key consequences for biological responses [79,80]. Thus, there has been a great deal of interest to illuminate the mechanisms of versatile actions of SnoN. SnoN complexes with R-Smad2, R-Smad3 and Smad4 [81,82]. Initial structural studies proposed a model whereby SnoN may lead to dissociation of R-Smad-Smad4 complex, offering a mechanism to explain negative role of SnoN on TGFβ signaling [77]. However, findings from a recent study suggest that SnoN can form a ternary complex with R-Smad2/3 and Smad4 multiprotein complex [81]. SnoN associates with other proteins including chromatin remodellers like the Histone deacetylases HDACs, which can be recruited to promoters of TGFβ-responsive genes [83]. The diverse functions of SnoN have raised the key question as to how SnoN actions are regulated. Interestingly, SnoN is a SUMO target and Lysines 50 and 383, which reside within SUMO consensus motifs, are major sites of SUMOylation in SnoN. PIAS1 and Transcription intermediary factor 1γ (TIF1γ) have been identified as two distinct SUMO E3 ligases that promote the SUMOylation of SnoN (Figure 3 and Figure 4) [14,58]. SUMOylation is important for SnoN to suppress TGFβ-induced EMT in NMuMG cell-derived organoids [58]. Overall, studies have revealed that each of PIAS1 and TIF1γ act via SnoN SUMOylation to suppress TGFβ-induced EMT in two-dimensional or three-dimensional culture systems [14,58] (Figure 3 and Figure 4). Data also suggest that the PIAS1-SnoN SUMOylation axis suppresses TGFβ-induced invasive growth of 3D-breast cancer cell-derived organoid system [59] (Figure 3 and Figure 4). Consistently, PIAS1 acts in a SUMO-E3 ligase-dependent manner to suppress the rate of breast-cancer cell-derived metastatic growth in a xenograft model [74]. In order to promote EMT and invasive growth, data suggest that TGFβ, in turn, reduces the protein abundance of PIAS1 and proportion of SUMOylated SnoN in non-transformed mammary epithelial cells and breast carcinomas [14,59]. These data thus suggest the existence of an interplay between TGFβ signaling and PIAS1-SnoN SUMOylation axis in controlling EMT and potentially cancer invasion and metastasis. Interestingly, the protein abundance and nuclear localization of PIAS1 were found to predict positive outcome in a cohort of breast cancer patients suggesting potential utility of these two PIAS1 parameters as prognostic biomarkers in breast cancer [59]. In a tissue microarray (TMA) study, investigating the protein levels of TIF1γ and SnoN in tumor tissue derived from bladder cancer patients and as compared to surrounding normal tissue, a reduction in the protein abundance of only TIF1γ was found [84]. Interestingly, the ability of overexpressed TIF1γ to suppress TGFβ-induced EMT and invasion appeared to be dependent positively on the expression status of SnoN in bladder cancer cell lines. The authors have also provided evidence suggesting that TIF1γ promotes the SUMOylation of SnoN to suppress TGFβ-induced EMT in bladder cancer cells. Future studies would be important to test this idea further including performing experiments to evaluate if alteration in the level or SUMO E3 ligase activity of TIF1γ affects the ability of SnoN to suppress TGFβ-induced EMT in bladder cancer cells [84]. Altogether, these data point to the importance of investigating the correlation between PIAS1, TIF1γ and SnoN in suppressing TGFβ-induced EMT and cancer invasiveness. 

## 6. EMT-TFs as Targets of the SUMO Pathway

### 6.1. Snail

Snail/Snai1, a zinc-finger containing protein, is an EMT-TF which has a short half-life of 20 to 45 minutes [85]. However, its levels can rapidly increase in response to EMT-inducing stimuli such as TGFβ [86,87]. In breast and prostate cancer cells, TβRI can be cleaved releasing an intra-cellular domain (ICD), which has been suggested to translocate into the nucleus and promote expression of genes, including Snail, that leads to increased cell migration [88,89]. Incubation of prostate and breast carcinoma cells with exogenous TGFβ was suggested to promote the assembly of TβRI ICD-Snail complexes that, in turn, upregulated the expression of TβRI. TGFβ was found to promote the SUMOylation of Snail on Lysine 234 and potentially stabilize and promote this transcription factor’s nuclear localization. Ectopic expression of a SUMO loss of function Snail in which Lysine 234 is converted to arginine was reported to reduce the ability of TGFβ to induce migration and invasion of prostate cancer cells as compared to wild-type Snail-expressing cells (Figure 3). SUMOylation was suggested to promote c-Jun-Snail interaction and responsive-gene expression [60]. Further studies are required to identify the SUMO E3 ligase that promotes Snail SUMOylation to provide a possible target in suppressing TGFβ-induced EMT. Interestingly, the TβRI-ICD retains the SUMO consensus lysine residue and which has been shown to be a target for SUMOylation [60]. Whether the SUMOylation of the TβRI alters its cleavage and subsequent activity with Snail remains to be investigated.

### 6.2. Slug

Slug/Snai2 is another zinc finger containing EMT-TF induced by TGFβ [86]. ARF (alternate reading frame protein product of the CDKN2A locus) protein was found to induce the SUMOylation of Slug at Lysine 192, and it potentially lead to increased migration in prostate cancer cells [61]. SUMOylation was found to increase the protein half-life of Slug and its ability to suppress E-cadherin expression [61] (Figure 3). ARF expression has been implicated to promote SUMOylation of multiple proteins including mouse double minute 2 (MdM2), although whether it is a SUMO E3 ligase is not well understood [90]. The protein TRIM28 has been suggested to be a SUMO E3 ligase associated with ARF-induced SUMOylation of the nucleolar protein nucleophosmin 1 (NPM1) [91]. TRIM28 has been suggested to promote TGFβ-induced EMT and invasiveness in lung and breast cancer cells [92,93]. The role of ARF protein in EMT and tumorigenesis remains poorly understood. Whether TRIM28 is the E3 ligase for Slug/Snai2 remains to be investigated.

### 6.3. Zeb2

The Zinc finger E-box-binding homeobox 2 (Zeb2), also known as Smad interacting protein 1 (SIP1), is a TGFβ target gene that acts as a repressor for E-cadherin gene expression [94]. SIP1 was found to be a part of the CtBP repressor complex which promotes histone repressive marks at the E-cadherin promoter causing reduced expression of E-cadherin transcript which is critical for EMT induction [95]. SIP1 can be SUMOylated at Lysines 391 and 866 in the repression domain which is promoted by the SUMO E3 ligase polycomb protein 2 (Pc2). Although, SUMOylation does not affect the subcellular localization of SIP1, the Lysine 391 and 866 to arginine double mutant displayed increased ability to suppress E-cadherin expression and thus promote EMT. It was found that SUMOylation suppresses SIP1 association with CtBP and hence repression of E-cadherin gene expression. Thus, SUMOylation may be a cellular mechanism that regulates SIP1-mediated EMT [62] 

## 7. Summary and Future Perspective

Protein SUMOylation is a rapidly expanding field with novel substrates and regulators being discovered on a regular basis. SUMOylation is a tightly controlled process and aberrations have been implicated in various diseases including cardiac, neurodegenerative, and malignant diseases [12,22,96]. The effect of dysregulation of the SUMO pathway in different cancers has recently been reviewed by Seeler and Dejean [22]. In particular, several studies have found that the protein abundance of specific enzymes responsible for SUMO conjugation and deconjugation can be altered in various tumor types which appear to be correlated either positively or negatively with patient outcome [22]. However, it was suggested that as the SUMO system is essential in all cell types, global alterations in the SUMO enzymes in any disease is a rare and isolated phenomenon [22]. Thus, the complex interplay between components of SUMO system and specific SUMO substrates appears to be a point of regulation in normal and tumor cells.

Recent findings point to an important interplay between the SUMO system and the TGFβ signaling pathway with implications for cellular processes including the fundamental process of EMT (See Table 1 for a summary). Members of the TGFβ signaling axis, effector proteins and regulators may be modified by the SUMO system. Conjugation of SUMO to a protein can alter the function, localization and stability of a given substrate, and often in a cell-type and context dependent manner. In turn, the TGFβ pathway has been found to positively or negatively affect the SUMO system, with potential implications for the specific SUMO substrates (Figure 3). Thus, the interplay may provide an on/off switch that may selectively affect specific types of biological outcomes in cells, tissue and organs. 

### 7.1. Global Analyses of SUMO System-TGFβ Signaling Interplay

The diverse SUMO pathway substrates with implications for TGFβ-induced EMT, discussed in this review, have been largely discovered and studied in isolation to answer how TGFβ signaling and responses can be regulated. EMT is a complex multi-step process involving the simultaneous or sequential alteration in stability, localization or function of a multitude of molecules including SUMO protein substrates, some of which have been the subject of this review. Thus, a direction for future studies would be to address the interplay between the various substrates as it relates to degree of SUMOylation and effect on TGFβ-induced EMT. Using loss or gain of function analyses, the epistatic relationship between different SUMO substrates with function in EMT can be determined. It will also be important to study the temporal and/or spatial determinants that control the ability of these diverse substrates to be targeted by the SUMO system and hence regulate EMT. These studies may help uncover novel mechanisms that may explain how the TGFβ pathway and SUMO system intersect. 

Global changes in DNA methylation during EMT in ovarian cancer cells have recently been evaluated which suggest that exogenous TGFβ stimulation may lead to significant changes in CpG island methylation of genes coding for proteins associated with EMT, survival and cancer progression [97]. On the other hand, global changes in protein SUMOylation have been studied in response to diverse external and internal stresses including heat [98], nutrient [99], DNA damage [100] and oxidative stress [101,102]. Thus, future studies can be designed to compare the global SUMOylation status in untreated versus TGFβ-stimulated cancer cell lines or patient-derived cells, representing a wide spectrum of cancer types. These types of studies, which can include differential labeling of untreated versus TGFβ-treated cells, affinity purification of SUMOylated proteins, followed by mass spectrometric analyses, would begin to address the role of the interplay between the SUMOylation machinery and TGFβ-signaling in controlling EMT induction and cancer progression.

As reviewed by Eifler and Vertegaal [103], several methods have been used to study proteome-based SUMOylation patterns. Conventional mass spectrometry-based analyses of SUMO conjugations are challenging due to many factors including low levels of SUMOylated proteins at any instance in part because of released SUMO protease activity upon cells lysis, and inefficient detection of large C-terminal tryptic fragments of SUMO (32 amino acids for SUMO2/3 and 19 for SUMO1) [103]. Endogenous SUMOylated proteins are identified either by using a SUMO antibody to enrich for SUMO-conjugated targets followed by MS/MS analyses [104] or by overexpressing a SIM-containing protein as a bait, like ring finger protein 4 (RNF4), to bind to multi or poly-SUMOylated chains followed by the bait immunopreciptations and MS/MS analyses of the immunocomplexes [105]. MS/MS analyses of SUMO immunocomplexes derived from lysates of SENP-inhibitor-treated cells transfected with vector control or one expressing a SUMO family member have also been used to identify novel SUMO-modified proteins [106]. In this context, overexpressing the SUMO proteins with specific point mutation to lead to shorter SUMO fragments on tryptic digestion and thus efficient analyses have been utilized in some of these studies [106,107]. Overall, these approaches can help identify global SUMOylation status in response to TGFβ stimulation of cells. Unravelling global SUMOylation patterns in patient samples may be another area of research with potential diagnostic and therapeutic implications. Using MS/MS analyses, recent studies have successfully evaluated the post-translational modifications in plasma-derived proteins and histones from pathology-derived tissues [108,109]. It would be interesting to determine if such approaches can be used to identify the potential role of SUMOylation and TGFβ signaling status as diagnostic biomarkers. 

### 7.2. Therapeutic Targeting of the SUMO Pathway

The role of TGFβ-induced EMT in diseases including cancer [26], points to the importance of developing EMT-targeting therapeutics. In this regard, the SUMO pathway may provide a target by which EMT can be potentially suppressed. Anacardic and ginkgolic acids are natural plant-based compounds which have been shown to specifically bind the SUMO E1 activating enzyme, leading to inhibition of SUMO conjugation [110]. Interestingly, anacardic acid has been suggested to display anticancer effects in different tumor types including breast, prostate and acute myeloid leukemia [111,112,113]. However, anacardic acid has also been reported to promote the proliferation of ovarian cancer cells [114]. Like anacardic acid, the recently characterized compound N106 has been reported to bind to E1. However, unlike anacardic acid, N106 was found to enhance the ability of the SUMO E1 activating enzyme to associate and activate SUMO [115]. Thus, it would be interesting to compare the effects of anacardic and ginkgolic acids to that of N106 in regulating TGFβ-induced EMT in 3D and other cellular model systems. Upregulation of specific SENPs have been implicated in promoting tumorigenesis and EMT in many cancer types including prostate, colon and thyroid cancers [22,116]. Thus, another area of research has focussed on developing specific inhibitors of SENPs [116,117]. 

It must be kept in mind that pharmacological inhibition of global SUMOylation may lead to unforeseeable risks given the dynamic nature of SUMO modification of different substrates. As SUMO modification has been suggested to lead to diverse and sometimes antagonistic effects on specific substrates with implications for TGFβ-induced EMT, it will be important to consider a variety of factors, including cell type, cell context, and identity of the SUMO substrate, when designing effective ways to modulate specific cellular processes in normal and diseased states. In addition, system-based high-throughput analyses of SUMO altering pharmaceutical agents can be performed, to begin understanding the effect of this class of drugs might have in a whole organism. Another avenue of research would be to identify small peptide-based drugs which may mimic the enzyme binding sites of specific substrates and thus may sequester specific SUMO E3 ligases or SENPs, relieving the effect on particular substrates. Finally, unbiased screening of different pharmacological libraries of small molecules or peptides may help identify different SUMO inhibitory or promoting compounds which then may be engineered to selectively alter specific SUMO pathways.

In the last 20 years, since the start of the identification of the SUMO pathway, we are only beginning to understand the vast impact of this post-translational modification in homeostasis and disease. With growing evidences implicating the role of SUMO modification on regulating TGFβ signaling axes and responses including EMT, future studies should focus on elucidating the molecular mechanisms mediating the ability of SUMOylated substrates to control biochemical and biological responses as well as how these modifications are regulated in cells. Such knowledge should help in the design and development of novel anticancer therapeutics.

## Figures and Tables

**Table 1:** Effect of SUMOylation on mediators and regulators of TGFβ-induced EMT and cancer cell invasion and migration.

SUMO Substrate	Effect on Transcriptional Responses	Effect on Biological Responses	References
TGFβ Receptor I	Not reported	Promotes TGFβ-induced invasion and lung metastasis of Ras-transformed fibroblasts.	[44]
Smad 3 and 4	Positive or negative in a cell and context dependent manner	Not reported	[50,51,52,53,54]
Smurf2	Not reported	Supresses TGFβ-induced EMT and invasive growth in non-transformed and transformed mammary cells respectively.	[56,57]
SnoN	Supresses TGFβ-induced gene expression in multiple cell types	Supresses TGFβ-induced EMT and invasive growth in non-transformed and transformed mammary cells, respectively. Similar effect may occur in bladder cancer.	[14,58,59,84]
Snail	Promotes c-Jun-Snail complex induced gene expression in different cancer cells.	Promotes TGFβ-induced migration and invasion of prostate and breast cancer cells.	[60]
Slug	Not reported	Promotes TGFβ-induced migration and invasion of prostate cancer cells.	[61]
Zeb2	Suppresses ability to bind to E-cadherin promoter.	Supresses EMT but effect on migration and invasion of tumor cells needs further analyses.	[62]

## References

[1] Kholodenko B.N. (2006). Cell-signalling dynamics in time and space. Nat. Rev. Mol. Cell Biol..

[2] Santos A.L., Lindner A.B. (2017). Protein posttranslational modifications: Roles in aging and age-related disease. Oxid. Med. Cell. Longev..

[3] Karve T.M., Cheema A.K. (2011). Small changes huge impact: The role of protein posttranslational modifications in cellular homeostasis and disease. J. Amino Acids.

[4] Duan G., Walther D. (2015). The roles of post-translational modifications in the context of protein interaction networks. PLoS Comput. Biol..

[5] Bettermann K., Benesch M., Weis S., Haybaeck J. (2012). SUMOylation in carcinogenesis. Cancer Lett..

[6] Dohmen R.J. (2004). SUMO protein modification. Biochim. Biophys. Acta.

[7] Palvimo J.J. (2007). Pias proteins as regulators of small ubiquitin-related modifier (SUMO) modifications and transcription. Biochem. Soc. Trans..

[8] Bohren K.M., Nadkarni V., Song J.H., Gabbay K.H., Owerbach D. (2004). A M55V polymorphism in a novel SUMO gene (SUMO-4) differentially activates heat shock transcription factors and is associated with susceptibility to type Ι diabetes mellitus. J. Biol. Chem..

[9] Liang Y.C., Lee C.C., Yao Y.L., Lai C.C., Schmitz M.L., Yang W.M. (2016). SUMO5, a novel poly-SUMO isoform, regulates PML nuclear bodies. Sci. Rep..

[10] Seeler J.S., Dejean A. (2003). Nuclear and unclear functions of SUMO. Nat. Rev. Mol. Cell Biol..

[11] Hendriks I.A., Vertegaal A.C. (2016). A comprehensive compilation of SUMO proteomics. Nat. Rev. Mol. Cell Biol..

[12] Anderson D.B., Zanella C.A., Henley J.M., Cimarosti H. (2017). SUMOylation: Implications for neurodegenerative diseases. Adv. Exp. Med. Biol..

[13] Johnson E.S. (2004). Protein modification by SUMO. Annu. Rev. Biophys. Biomol..

[14] Netherton S.J., Bonni S. (2010). Suppression of TGFβ-induced epithelial-mesenchymal transition like phenotype by a PIAS1 regulated SUMOylation pathway in NMuMG epithelial cells. PLoS ONE.

[15] Vertegaal A.C. (2010). SUMO chains: Polymeric signals. Biochem. Soc. Trans..

[16] Eisenhardt N., Chaugule V.K., Koidl S., Droescher M., Dogan E., Rettich J., Sutinen P., Imanishi S.Y., Hofmann K., Palvimo J.J. (2015). A new vertebrate SUMO enzyme family reveals insights into SUMO-chain assembly. Nat. Struct. Mol. Biol..

[17] Hecker C.M., Rabiller M., Haglund K., Bayer P., Dikic I. (2006). Specification of SUMO1-and SUMO2-interacting motifs. J. Biol. Chem..

[18] Rabellino A., Andreani C., Scaglioni P.P. (2017). The role of pias SUMO E3-ligases in cancer. Cancer Res..

[19] Pichler A., Gast A., Seeler J.S., Dejean A., Melchior F. (2002). The nucleoporin RanBP2 has SUMO1 E3 ligase activity. Cell.

[20] Chu Y., Yang X. (2011). SUMO E3 ligase activity of trim proteins. Oncogene.

[21] Kagey M.H., Melhuish T.A., Wotton D. (2003). The polycomb protein Pc2 is a SUMO E3. Cell.

[22] Seeler J.S., Dejean A. (2017). SUMO and the robustness of cancer. Nat. Rev. Cancer.

[23] Creighton C.J., Gibbons D.L., Kurie J.M. (2013). The role of epithelial-mesenchymal transition programming in invasion and metastasis: A clinical perspective. Cancer Manag. Res..

[24] Miettinen P.J., Ebner R., Lopez A.R., Derynck R. (1994). TGF-beta induced transdifferentiation of mammary epithelial cells to mesenchymal cells: Involvement of type i receptors. J. Cell Biol..

[25] Lamouille S., Xu J., Derynck R. (2014). Molecular mechanisms of epithelial-mesenchymal transition. Nat. Rev. Mol. Cell Biol..

[26] De Craene B., Berx G. (2013). Regulatory networks defining emt during cancer initiation and progression. Nat. Rev. Cancer.

[27] Derynck R., Muthusamy B.P., Saeteurn K.Y. (2014). Signaling pathway cooperation in TGF-beta-induced epithelial-mesenchymal transition. Curr. Opin. Cell Biol..

[28] Nieto M.A. (2011). The ins and outs of the epithelial to mesenchymal transition in health and disease. Annu. Rev. Cell Dev. Biol..

[29] Thiery J.P. (2002). Epithelial-mesenchymal transitions in tumour progression. Nat. Rev. Cancer.

[30] Mani S.A., Guo W., Liao M.J., Eaton E.N., Ayyanan A., Zhou A.Y., Brooks M., Reinhard F., Zhang C.C., Shipitsin M. (2008). The epithelial-mesenchymal transition generates cells with properties of stem cells. Cell.

[31] Morel A.P., Lievre M., Thomas C., Hinkal G., Ansieau S., Puisieux A. (2008). Generation of breast cancer stem cells through epithelial-mesenchymal transition. PLoS ONE.

[32] Medema J.P. (2013). Cancer stem cells: The challenges ahead. Nat. Cell Biol..

[33] Robson E.J., Khaled W.T., Abell K., Watson C.J. (2006). Epithelial-to-mesenchymal transition confers resistance to apoptosis in three murine mammary epithelial cell lines. Differentiation.

[34] Katsuno Y., Lamouille S., Derynck R. (2013). TGF-beta signaling and epithelial-mesenchymal transition in cancer progression. Curr. Opin. Oncol..

[35] Blobe G.C., Schiemann W.P., Lodish H.F. (2000). Role of transforming growth factor beta in human disease. N. Engl. J. Med..

[36] Biernacka A., Dobaczewski M., Frangogiannis N.G. (2011). TGF-beta signaling in fibrosis. Growth Factors.

[37] Lebrun J.J. (2012). The dual role of TGFβ in human cancer: From tumor suppression to cancer metastasis. ISRN Mol. Biol..

[38] Itoh S., Itoh F., Goumans M.J., Ten Dijke P. (2000). Signaling of transforming growth factor-beta family members through Smad proteins. Eur. J. Biochem..

[39] Shi Y., Massague J. (2003). Mechanisms of TGF-beta signaling from cell membrane to the nucleus. Cell.

[40] Derynck R., Akhurst R.J., Balmain A. (2001). TGF-beta signaling in tumor suppression and cancer progression. Nat. Genet..

[41] Massague J. (2008). TGFβ in cancer. Cell.

[42] Miyazawa K., Miyazono K. (2017). Regulation of TGF-beta family signaling by inhibitory Smads. Cold Spring Harb. Perspect. Biol..

[43] O’Connor J.W., Gomez E.W. (2014). Biomechanics of TGFβ-induced epithelial-mesenchymal transition: Implications for fibrosis and cancer. Clin. Trans. Med..

[44] Kang J.S., Saunier E.F., Akhurst R.J., Derynck R. (2008). The type Ι TGF-beta receptor is covalently modified and regulated by SUMOylation. Nat. Cell Biol..

[45] Hietakangas V., Anckar J., Blomster H.A., Fujimoto M., Palvimo J.J., Nakai A., Sistonen L. (2006). Pdsm, a motif for phosphorylation-dependent SUMO modification. Proc. Natl. Acad. Sci. USA.

[46] Chen T., Carter D., Garrigue-Antar L., Reiss M. (1998). Transforming growth factor beta type Ι receptor kinase mutant associated with metastatic breast cancer. Cancer Res..

[47] Chen T., Yan W., Wells R.G., Rimm D.L., McNiff J., Leffell D., Reiss M. (2001). Novel inactivating mutations of transforming growth factor-beta type Ι receptor gene in head-and-neck cancer metastases. Int. J. Cancer.

[48] Macias M.J., Martin-Malpartida P., Massague J. (2015). Structural determinants of Smad function in TGF-beta signaling. Trends Biochem. Sci..

[49] Burch M.L., Zheng W., Little P.J. (2011). Smad linker region phosphorylation in the regulation of extracellular matrix synthesis. Cell Mol. Life Sci..

[50] Lee P.S., Chang C., Liu D., Derynck R. (2003). SUMOylation of Smad4, the common Smad mediator of transforming growth factor-beta family signaling. J. Biol. Chem..

[51] Lin X., Liang M., Liang Y.Y., Brunicardi F.C., Melchior F., Feng X.H. (2003). Activation of transforming growth factor-beta signaling by SUMO-1 modification of tumor suppressor Smad4/DPC4. J. Biol. Chem..

[52] Long J., Wang G., He D., Liu F. (2004). Repression of Smad4 transcriptional activity by SUMO modification. Biochem. J..

[53] Ohshima T., Shimotohno K. (2003). Transforming growth factor-beta-mediated signaling via the p38 MAP kinase pathway activates Smad-dependent transcription through SUMO-1 modification of Smad4. J. Biol. Chem..

[54] Lin X., Liang M., Liang Y.Y., Brunicardi F.C., Feng X.H. (2003). SUMO-1/Ubc9 promotes nuclear accumulation and metabolic stability of tumor suppressor Smad4. J. Biol. Chem..

[55] Imoto S., Sugiyama K., Muromoto R., Sato N., Yamamoto T., Matsuda T. (2003). Regulation of transforming growth factor-beta signaling by protein inhibitor of activated stat, piasy through Smad3. J. Biol. Chem..

[56] Chandhoke A.S., Karve K., Dadakhujaev S., Netherton S., Deng L., Bonni S. (2016). The ubiquitin ligase Smurf2 suppresses TGFβ-induced epithelial-mesenchymal transition in a SUMOylation-regulated manner. Cell Death Differ..

[57] Chandhoke A.S., Chanda A., Karve K., Deng L., Bonni S. (2017). The PIAS3-Smurf2 SUMOylation pathway suppresses breast cancer organoid invasiveness. Oncotarget.

[58] Ikeuchi Y., Dadakhujaev S., Chandhoke A.S., Huynh M.A., Oldenborg A., Ikeuchi M., Deng L., Bennett E.J., Harper J.W., Bonni A. (2014). TIF1γ protein regulates epithelial-mesenchymal transition by operating as a small ubiquitin-like modifier (SUMO) E3 ligase for the transcriptional regulator SnoN1. J. Biol. Chem..

[59] Chanda A., Chan A., Deng L., Kornaga E.N., Enwere E.K., Morris D.G., Bonni S. (2017). Identification of the SUMO E3 ligase PIAS1 as a potential survival biomarker in breast cancer. PLoS ONE.

[60] Gudey S.K., Sundar R., Heldin C.H., Bergh A., Landstrom M. (2017). Pro-invasive properties of Snail1 are regulated by SUMOylation in response to TGFβ stimulation in cancer. Oncotarget.

[61] Xie Y., Liu S., Lu W., Yang Q., Williams K.D., Binhazim A.A., Carver B.S., Matusik R.J., Chen Z. (2014). Slug regulates E-cadherin repression via p19Arf in prostate tumorigenesis. Mol. Oncol..

[62] Long J., Zuo D., Park M. (2005). Pc2-mediated SUMOylation of Smad-interacting protein 1 attenuates transcriptional repression of e-cadherin. J. Biol. Chem..

[63] Zhou X., Gao C., Huang W., Yang M., Chen G., Jiang L., Gou F., Feng H., Ai N., Xu Y. (2014). High glucose induces SUMOylation of Smad4 via SUMO2/3 in mesangial cells. BioMed Res. Int..

[64] Hsu W.L., Ma Y.L., Liu Y.C., Lee E.H.Y. (2017). Smad4 SUMOylation is essential for memory formation through upregulation of the skeletal myopathy gene tpm2. BMC Biol..

[65] Xiu D., Wang Z., Cui L., Jiang J., Yang H., Liu G. (2018). SUMOylation of Smad 4 ameliorates the oxidative stress-induced apoptosis in osteoblasts. Cytokine.

[66] Long J., Wang G., Matsuura I., He D., Liu F. (2004). Activation of Smad transcriptional activity by protein inhibitor of activated STAT3 (PIAS3). Proc. Natl. Acad. Sci. USA.

[67] Long J., Matsuura I., He D., Wang G., Shuai K., Liu F. (2003). Repression of Smad transcriptional activity by piasy, an inhibitor of activated stat. Proc. Natl. Acad. Sci. USA.

[68] Zhu H., Kavsak P., Abdollah S., Wrana J.L., Thomsen G.H. (1999). A Smad ubiquitin ligase targets the bmp pathway and affects embryonic pattern formation. Nature.

[69] Lin X., Liang M., Feng X.H. (2000). Smurf2 is a ubiquitin E3 ligase mediating proteasome-dependent degradation of Smad2 in transforming growth factor-beta signaling. J. Biol. chem..

[70] Inoue Y., Imamura T. (2008). Regulation of TGF-beta family signaling by E3 ubiquitin ligases. Cancer Sci..

[71] Kavsak P., Rasmussen R.K., Causing C.G., Bonni S., Zhu H., Thomsen G.H., Wrana J.L. (2000). Smad7 binds to Smurf2 to form an E3 ubiquitin ligase that targets the TGF beta receptor for degradation. Mol. Cell.

[72] Bonni S., Wang H.R., Causing C.G., Kavsak P., Stroschein S.L., Luo K., Wrana J.L. (2001). TGF-beta induces assembly of a Smad2-Smurf2 ubiquitin ligase complex that targets SnoN for degradation. Nat. Cell Biol..

[73] Debnath J., Brugge J.S. (2005). Modelling glandular epithelial cancers in three-dimensional cultures. Nat. Rev. Cancer.

[74] Dadakhujaev S., Salazar-Arcila C., Netherton S.J., Chandhoke A.S., Singla A.K., Jirik F.R., Bonni S. (2014). A novel role for the SUMO E3 ligase PIAS1 in cancer metastasis. Oncoscience.

[75] Wang L., Banerjee S. (2004). Differential pias3 expression in human malignancy. Oncol. Rep..

[76] Bonni S., Bonni A. (2012). SnoN signaling in proliferating cells and postmitotic neurons. FEBS Lett..

[77] Deheuninck J., Luo K. (2009). Ski and SnoN, potent negative regulators of TGF-beta signaling. Cell Res..

[78] Stroschein S.L., Wang W., Zhou S., Zhou Q., Luo K. (1999). Negative feedback regulation of TGF-beta signaling by the SnoN oncoprotein. Science.

[79] Ikeuchi Y., Stegmuller J., Netherton S., Huynh M.A., Masu M., Frank D., Bonni S., Bonni A. (2009). A SnoN-Ccd1 pathway promotes axonal morphogenesis in the mammalian brain. J. Neurosci..

[80] Sarker K.P., Wilson S.M., Bonni S. (2005). SnoN is a cell type-specific mediator of transforming growth factor-beta responses. J. Biol. Chem..

[81] Wallden K., Nyman T., Hallberg B.M. (2017). SnoN stabilizes the Smad3/Smad4 protein complex. Sci. Rep..

[82] Mizuide M., Hara T., Furuya T., Takeda M., Kusanagi K., Inada Y., Mori M., Imamura T., Miyazawa K., Miyazono K. (2003). Two short segments of Smad3 are important for specific interaction of Smad3 with c-Ski and SnoN. J. Biol. Chem..

[83] Nomura T., Khan M.M., Kaul S.C., Dong H.D., Wadhwa R., Colmenares C., Kohno I., Ishii S. (1999). Ski is a component of the histone deacetylase complex required for transcriptional repression by mad and thyroid hormone receptor. Genes Dev..

[84] Yin X., Xu C., Zheng X., Yuan H., Liu M., Qiu Y., Chen J. (2016). SnoN suppresses TGF-beta-induced epithelial-mesenchymal transition and invasion of bladder cancer in a tif1gamma-dependent manner. Oncol. Rep..

[85] De Herreros A.G., Peiro S., Nassour M., Savagner P. (2010). Snail family regulation and epithelial mesenchymal transitions in breast cancer progression. J. Mammary Gland Biol..

[86] Wang Y., Shi J., Chai K., Ying X., Zhou B.P. (2013). The role of Snail in emt and tumorigenesis. Curr. Cancer Drug Target.

[87] Zhou B.P., Deng J., Xia W., Xu J., Li Y.M., Gunduz M., Hung M.C. (2004). Dual regulation of Snail by gsk-3beta-mediated phosphorylation in control of epithelial-mesenchymal transition. Nat. Cell Biol..

[88] Mu Y., Sundar R., Thakur N., Ekman M., Gudey S.K., Yakymovych M., Hermansson A., Dimitriou H., Bengoechea-Alonso M.T., Ericsson J. (2011). Traf6 ubiquitinates TGFβ type Ι receptor to promote its cleavage and nuclear translocation in cancer. Nat. Commun..

[89] Chandra M., Zang S., Li H., Zimmerman L.J., Champer J., Tsuyada A., Chow A., Zhou W., Yu Y., Gao H. (2012). Nuclear translocation of type Ι transforming growth factor beta receptor confers a novel function in RNA processing. Mol. Cell Biol..

[90] Den Besten W., Kuo M.L., Tago K., Williams R.T., Sherr C.J. (2006). Ubiquitination of, and SUMOylation by, the Arf tumor suppressor. Israel Med. Assoc. J..

[91] Neo S.H., Itahana Y., Alagu J., Kitagawa M., Guo A.K., Lee S.H., Tang K., Itahana K. (2015). TRIM28 is an E3 ligase for Arf-mediated NPM1/B23 SUMOylation that represses centrosome amplification. Mol. Cell. Biol..

[92] Chen L., Munoz-Antonia T., Cress W.D. (2014). TRIM28 contributes to EMT via regulation of E-cadherin and N-cadherin in lung cancer cell lines. PLoS ONE.

[93] Wei C., Cheng J., Zhou B., Zhu L., Khan M.A., He T., Zhou S., He J., Lu X., Chen H. (2016). Tripartite motif containing 28 (TRIM28) promotes breast cancer metastasis by stabilizing TWIST1 protein. Sci. Rep..

[94] Comijn J., Berx G., Vermassen P., Verschueren K., van Grunsven L., Bruyneel E., Mareel M., Huylebroeck D., van Roy F. (2001). The two-handed E box binding zinc finger protein SIP1 downregulates E-cadherin and induces invasion. Mol. Cell.

[95] Shi Y., Sawada J., Sui G., Affar el B., Whetstine J.R., Lan F., Ogawa H., Luke M.P., Nakatani Y., Shi Y. (2003). Coordinated histone modifications mediated by a CtBP co-repressor complex. Nature.

[96] Mendler L., Braun T., Muller S. (2016). The ubiquitin-like SUMO system and heart function: From development to disease. Circ. Res..

[97] Cardenas H., Vieth E., Lee J., Segar M., Liu Y., Nephew K.P., Matei D. (2014). TGF-beta induces global changes in DNA methylation during the epithelial-to-mesenchymal transition in ovarian cancer cells. Epigenetics.

[98] Golebiowski F., Matic I., Tatham M.H., Cole C., Yin Y., Nakamura A., Cox J., Barton G.J., Mann M., Hay R.T. (2009). System-wide changes to SUMO modifications in response to heat shock. Sci. Signal..

[99] Yang W., Thompson J.W., Wang Z., Wang L., Sheng H., Foster M.W., Moseley M.A., Paschen W. (2012). Analysis of oxygen/glucose-deprivation-induced changes in SUMO3 conjugation using silac-based quantitative proteomics. J. Proteome Res..

[100] Psakhye I., Jentsch S. (2012). Protein group modification and synergy in the SUMO pathway as exemplified in DNA repair. Cell.

[101] Bossis G., Melchior F. (2006). Regulation of SUMOylation by reversible oxidation of SUMO conjugating enzymes. Mol. Cell.

[102] Xu Z., Lam L.S., Lam L.H., Chau S.F., Ng T.B., Au S.W. (2008). Molecular basis of the redox regulation of SUMO proteases: A protective mechanism of intermolecular disulfide linkage against irreversible sulfhydryl oxidation. FASEB J..

[103] Eifler K., Vertegaal A.C. (2015). Mapping the SUMOylated landscape. FEBS J..

[104] Becker J., Barysch S.V., Karaca S., Dittner C., Hsiao H.H., Berriel Diaz M., Herzig S., Urlaub H., Melchior F. (2013). Detecting endogenous SUMO targets in mammalian cells and tissues. Nat. Struct. Mol. Biol..

[105] Bruderer R., Tatham M.H., Plechanovova A., Matic I., Garg A.K., Hay R.T. (2011). Purification and identification of endogenous polySUMO conjugates. EMBO Rep..

[106] Hendriks I.A., D’Souza R.C., Yang B., Verlaan-de Vries M., Mann M., Vertegaal A.C. (2014). Uncovering global SUMOylation signaling networks in a site-specific manner. Nat. Struct. Mol. Biol..

[107] Tammsalu T., Matic I., Jaffray E.G., Ibrahim A.F.M., Tatham M.H., Hay R.T. (2014). Proteome-wide identification of SUMO2 modification sites. Sci. Signal..

[108] Petushkova N.A., Zgoda V.G., Pyatnitskiy M.A., Larina O.V., Teryaeva N.B., Potapov A.A., Lisitsa A.V. (2017). Post-translational modifications of FDA-approved plasma biomarkers in glioblastoma samples. PLoS ONE.

[109] Noberini R., Uggetti A., Pruneri G., Minucci S., Bonaldi T. (2016). Pathology tissue-quantitative mass spectrometry analysis to profile histone post-translational modification patterns in patient samples. Mol. Cell. Proteom..

[110] Fukuda I., Ito A., Hirai G., Nishimura S., Kawasaki H., Saitoh H., Kimura K., Sodeoka M., Yoshida M. (2009). Ginkgolic acid inhibits protein SUMOylation by blocking formation of the E1-SUMO intermediate. Chem. Biol..

[111] Bossis G., Sarry J.E., Kifagi C., Ristic M., Saland E., Vergez F., Salem T., Boutzen H., Baik H., Brockly F. (2014). The ROS/SUMO axis contributes to the response of acute myeloid leukemia cells to chemotherapeutic drugs. Cell Rep..

[112] Tan J., Chen B., He L., Tang Y., Jiang Z., Yin G., Wang J., Jiang X. (2012). Anacardic acid (6-pentadecylsalicylic acid) induces apoptosis of prostate cancer cells through inhibition of androgen receptor and activation of p53 signaling. Chin. J. Cancer Res..

[113] Schultz D.J., Muluhngwi P., Alizadeh-Rad N., Green M.A., Rouchka E.C., Waigel S.J., Klinge C.M. (2017). Genome-wide miRNA response to anacardic acid in breast cancer cells. PLoS ONE.

[114] Xiu Y.L., Zhao Y., Gou W.F., Chen S., Takano Y., Zheng H.C. (2014). Anacardic acid enhances the proliferation of human ovarian cancer cells. PLoS ONE.

[115] Kho C., Lee A., Jeong D., Oh J.G., Gorski P.A., Fish K., Sanchez R., DeVita R.J., Christensen G., Dahl R. (2015). Small-molecule activation of SERCA2a SUMOylation for the treatment of heart failure. Nat. Commun..

[116] Bogachek M.V., De Andrade J.P., Weigel R.J. (2015). Regulation of epithelial-mesenchymal transition through SUMOylation of transcription factors. Cancer Res..

[117] Kumar A., Zhang K.Y. (2015). Advances in the development of SUMO specific protease (SENP) inhibitors. Comput. Struct. Biotechnol..

